# Short and Long-Term Changes in Social Odor Recognition and Plasma Cytokine Levels Following Oxygen (^16^O) Ion Radiation Exposure

**DOI:** 10.3390/ijms20020339

**Published:** 2019-01-15

**Authors:** Carli B. Jones, Ami Mange, Lauren Granata, Benjamin Johnson, Robert D. Hienz, Catherine M. Davis

**Affiliations:** 1 Department of Pathology, Johns Hopkins University School of Medicine, Baltimore, MD 21287, USA; cjone22@jhu.edu; 2 Division of Behavioral Biology, Department of Psychiatry and Behavioral Sciences, Johns Hopkins University School of Medicine, Baltimore, MD 21224, USA; amange1@jhu.edu (A.M.); lgranat3@jhu.edu (L.G.); bjohn145@jhmi.edu (B.J.); bhienz@jhmi.edu (R.D.H.)

**Keywords:** social recognition, HZE ions, rats, memory, space radiation, cytokines

## Abstract

Future long-duration space missions will involve travel outside of the Earth’s magnetosphere protection and will result in astronauts being exposed to high energy and charge (HZE) ions and protons. Exposure to this type of radiation can result in damage to the central nervous system and deficits in numerous cognitive domains that can jeopardize mission success. Social processing is a cognitive domain that is important for people living and working in groups, such as astronauts, but it has received little attention in terms of HZE ion exposure. In the current study, we assessed the effects of whole-body oxygen ion (^16^O; 1000 MeV/n) exposure (1 or 10 cGy) on social odor recognition memory in male Long-Evans rats at one and six months following exposure. Radiation exposure did not affect rats’ preferences for a novel social odor experienced during Habituation at either time point. However, rats exposed to 10 cGy displayed short and long-term deficits in 24-h social recognition. In contrast, rats exposed to 1 cGy only displayed long-term deficits in 24-h social recognition. While an age-related decrease in Ki67+ staining (a marker of cell proliferation) was found in the subventricular zone, it was unaffected by radiation exposure. At one month following exposure, plasma KC/GRO (CXCL1) levels were elevated in the 1 cGy rats, but not in the 10 cGy rats, suggesting that peripheral levels of this cytokine could be associated with intact social recognition at earlier time points following radiation exposure. These results have important implications for long-duration missions and demonstrate that behaviors related to social processing could be negatively affected by HZE ion exposure.

## 1. Introduction

In the near future, astronauts will spend more prolonged periods in space, venturing to the moon and Mars. These missions will require travel outside of the Earth’s magnetosphere, where exposure to galactic cosmic radiation (GCR) and solar particle events (SPE) will occur [1]. Current spacecraft provide limited protection from GCR exposure, which includes alpha particles, high energy, charge (HZE) particles, and protons [2]. Exposure to these particles has already been shown to have deleterious effects on animals’ central nervous system (CNS), and thus could result in impaired neurobehavioral performances in humans, jeopardizing astronaut health and mission success [3,4,5,6,7,8,9,10,11,12,13,14,15,16,17]. For these reasons, NASA’s Integrated Research Plan includes studies to determine the effects of space radiation exposure on numerous body systems, including the CNS, and on various cognitive domains, such as memory and social processing. 

In order to address NASA’s questions regarding the effects of space radiation on humans, animal studies using charged particles, as opposed to electromagnetic radiation, are necessary. Even though HZE ions comprise a small portion of the total radiation dose hypothesized for an astronaut in deep space (~20–70 cGy), they are densely ionizing and produce distinct biological effects when compared to sparsely ionizing and low LET radiation, such as X-rays [1,2]. While numerous animal studies have shown that significant and long-term neurobehavioral impairments occur following exposure to protons and HZE ions [4,5,6,7,8,9,10,11,12,13,14,15,16,17], several cognitive domains important for astronaut health and well-being remain relatively unexplored. Social cognition is one such domain and is important for humans working in groups, especially in the confined and isolated environment of long-duration space flight. One of the most widely used tests to assess deficits in social cognition is the social preference/recognition memory test, where a subject rodent must differentiate between socially relevant objects (e.g., a live rodent and an inanimate object, two live rodents, or olfactory cues from different rodents) [18,19]. When allowed to freely explore these stimuli, rodents typically spend a greater percentage of time exploring novel stimuli during these tests and they also display a predictable pattern of habituation to these stimuli. For example, normal rodents interact more with an unfamiliar conspecific or the conspecific’s olfactory cues, as opposed to a familiar conspecific. There are several variations of this test, with the stimuli being live rodents or objects impregnated with the odors of conspecifics (e.g., soiled bedding, urine-soaked paper, wooden beads) [20]. We have used a variation of this test [21,22], termed the Social Odor Recognition Memory (SORM) test, where the subjects initially show exploration time differences between self-odors and a novel conspecific’s odor during “Habituation”, and an additional novel conspecific’s odor on the following “Recognition Test” [23,24,25]. Decreased preference for the first novel odor on the latter Recognition Test, experienced during Habituation 24-h earlier, is indicative of a social recognition memory deficit.

Our laboratory [22] and the Rosi laboratory [8] have recently shown strikingly similar deficits in social recognition in two different versions of the social recognition test with male rats and male mice, respectively, following 25 cGy ^16^O ion exposure (1000 or 600 MeV/n, respectively). More specifically, these studies showed that 25 cGy ^16^O ion exposure resulted in deficits in social recognition memory when measured at one month, four months, and six months post-exposure, suggesting a long-term deficit in social processing following HZE exposure. While we showed no radiation-induced change in Ki67+ staining in the subventricular zone (SVZ) at six months, Krukowski and colleagues [8] showed a significant correlation between social recognition memory and plasma immune cell levels, which the authors suggested as a possible biomarker of deficits in social processing. As a follow-up to these published reports, the current study exposed male Long-Evans rats to 1 or 10 cGy ^16^O ion radiation (1000 MeV/n) and then tested these rats for social odor recognition memory at both one and six months following exposure. Subsets of rats at each time point were used to assess plasma cytokine levels and Ki67+ staining in the SVZ. 

The goals of this study were to (1) determine if these lower doses of ^16^O ions result in short- and/or long-term deficits in social recognition, (2) determine if a threshold dose of ^16^O ions exists for inducing social recognition deficits, (3) determine if these changes are associated with alterations in cell proliferation in the SVZ at an earlier time point, and (4) assess changes in inflammatory cytokines that could be used as potential biomarkers indicative of deficits in social processing. 

## 2. Results

### 2.1. Habituation to the Novel 1 Odor is Unaffected by ^16^O Ion Exposure

**Figure 1:**
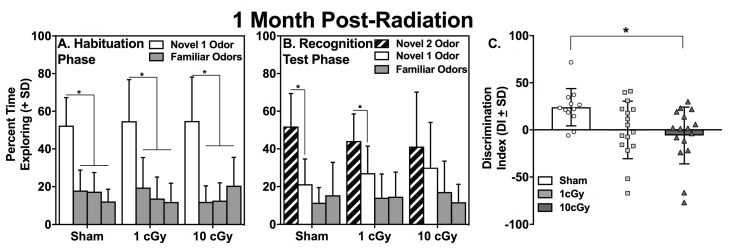
Mean percentage of exploration (+SD) of novel and familiar odors for sham (*n* = 12), 1 cGy (*n* = 16), and 10cGy (*n* = 16) ^16^O ion exposed rats on (**A**) Trial 1 of Habituation and (B) Trial 1 of the Recognition Test, one month following exposure. All rats displayed a significantly greater percentage of exploration of the novel 1 odor on Habituation compared to the familiar odors (* *p* < 0.05). Sham and 1 cGy-exposed rats displayed significantly greater exploration of the novel 2 odor during the Recognition Test (* *p* ≤ 0.05). Each group of bars in **A** and **B** represents mean percentage of exploration of the four odor-impregnated wooden beads during either Habituation or the Recognition Test. Solid white bars: novel 1 odor. Solid gray bars: familiar odors. Hatched bars: novel 2 odor. (**C**) The 10 cGy-exposed rats displayed a discrimination index significantly less than the sham rats (* *p* < 0.05); bars represent group averages and symbols represent individual animals.

**Figure 2:**
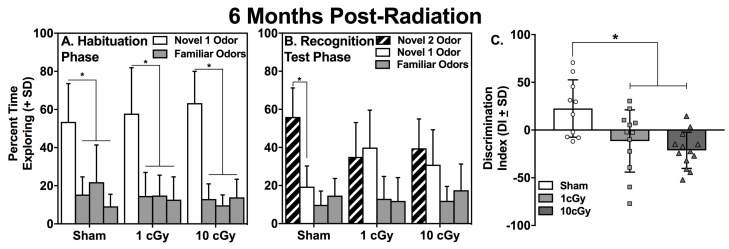
Mean percentage of exploration of novel and familiar odors for sham (*n* = 10), 1 cGy (*n* = 13), and 10cGy (*n* = 13) ^16^O ion exposed rats on (**A**) Trial 1 of Habituation and (B) Trial 1 of the Recognition Test at six months following exposure. All rats displayed significantly greater percentage of exploration of the novel 1 odor on Habituation compared to the familiar odors (* *p* ≤ 0.05). Only sham control rats displayed significantly greater exploration of the novel 2 odor during the Recognition Test (* *p* ≤ 0.05). Each group of bars in A and B represents mean percentage of exploration of the four odor-impregnated wooden beads during either Habituation or the Recognition Test. Solid white bars: novel odor 1. Solid gray bars: familiar odors. Hatched bars: novel odor 2. (**C**) The 1 and 10 cGy-exposed rats displayed discrimination indices significantly less than the sham control rats (* *p* ≤ 0.05); bars represent group averages and symbols represent individual animals.

During the Habituation phase of the social odor recognition memory test (SORM), rats explore wooden beads impregnated with their own odors (i.e., familiar odors) or the odors of a novel conspecific rat (novel 1 odor). Rats with intact social odor discrimination and processing will spend a greater percentage of time exploring the novel 1 odor (N1 odor) compared to the familiar odors. Sham (*n* = 12), 1 cGy (*n* = 16), and 10 cGy (*n* = 16) exposed rats all displayed intact social odor discrimination during this phase, as evidenced by having spent a significantly greater amount of time exploring the N1 odor (Figure 1, left panel) compared to all three familiar odor beads during the first trial of Habituation (all *p*’s ≤ 0.002; see Table 1), when assessed at one month following radiation exposure. Rats with intact social odor discrimination also show habituation during the three trials of this phase, which is indicated by significantly less exploration of the N1 odor on the third trial compared to the first trial. All groups showed habituation to the N1 odor over trials, which was illustrated by significantly decreased exploration of the N1 odor on the third trial compared to the same N1 odor on the first trial (all *p*’s ≤ 0.024; see Table 1). A similar pattern of exploration was found at six months post-exposure, with sham (*n* = 10), 1 cGy (n = 13), and 10 cGy (n = 13) groups displaying significantly greater exploration of the N1 odor bead on the first trial compared to the familiar odor beads (all *p*’s ≤ 0.005; see Figure 2, left panel, and Table 2) and significantly greater exploration of the N1 odor on trial 1 compared to trial 3 (all *p*’s ≤ 0.024; see Table 2). 

### 2.2. Social Recognition is Dose-Dependently Impaired at One Month, but Not at Six Months, Following ^16^O Ion Exposure

At one month following radiation exposure, both the sham- and 1 cGy-irradiated rats displayed significantly greater percentages of time exploring the N2 bead compared to the N1 bead on Trial 1 of the Recognition Test (all *p’*s ≤ 0.016, Cohen’s *d* = 1.15 and 0.68, for sham and 1 cGy, respectively). In contrast, the 10 cGy-irradiated animals explored the N2 and N1 beads to a similar extent (*p* = 0.362) at this early time point (see Figure 1, middle panel). The one-way ANOVA evaluating the discrimination indices at this time point was significant (F(2,40) = 3.939, *p =* 0.0274) and further supported the social recognition deficit in the 10 cGy-exposed rats (Figure 1, right panel); only this group displayed a discrimination index that was significantly lower than the sham control rats (*p =* 0.0194). 

At six months following radiation exposure, only the sham-irradiated rats displayed a significantly greater percentage of time exploring the N2 bead compared to the N1 bead on Trial 1 of the Recognition Test (*p* < 0.001, Cohen’s *d* = 1.679). Both irradiated groups displayed similar percentages of time exploring the N2 and N1 beads on the Recognition Test day (all *p’*s ≥ 0.316; see Figure 2, middle panel). The one-way ANOVA comparing the discrimination indices at this time point was significant (F(2,32) = 7.624, *p* = 0.002) and further supported the social recognition deficits in both the 1 and 10 cGy-exposed rats (Figure 2, right panel). Both groups had discrimination indices that were significantly lower than the sham control rats (*p’s* ≤ 0.013). Levene’s Test for homogeneity of variances was not significant at one month or at six months for the ANOVAs comparing the discrimination indices (*p* = 0.267 and 0.282, respectively).

**Figure 3:**
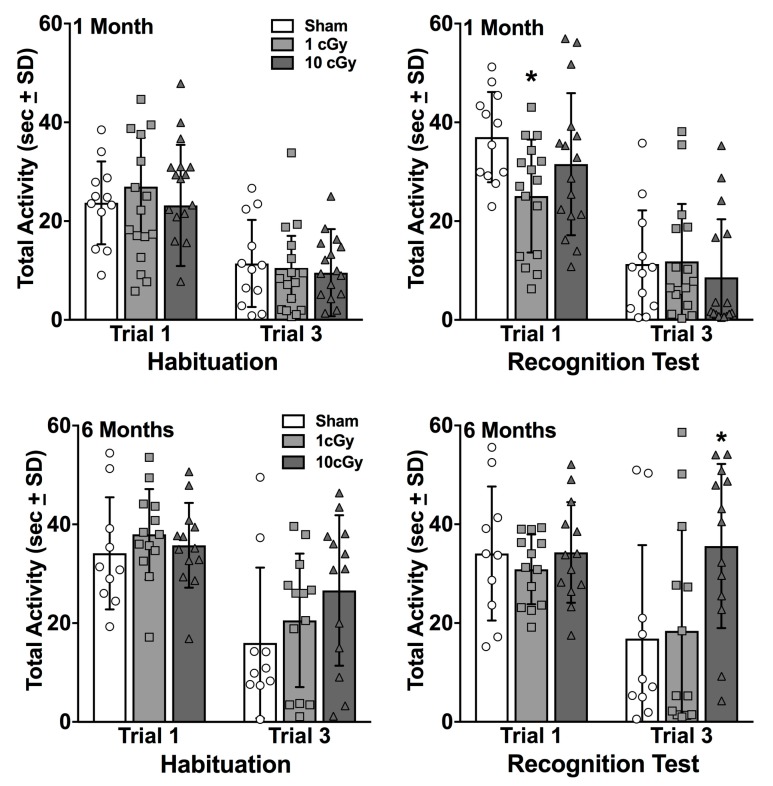
Mean total seconds of exploration on Trials 1 and 3 for Habituation and the Recognition Test at one month (top panels; *n* = 12–16) and six months (bottom panels; *n* = 10–13) following exposure. No differences were found among the groups during Habituation at either time point. At one month following exposure, 1 cGy rats had significantly less exploration on Trial 1 of the Recognition Test compared to sham control rats (* *p* ≤ 0.05). At six months, the 10 cGy rats had significantly greater exploration on Trial 3 of the Recognition Test compared to both sham controls and 1 cGy exposed rats (* *p* ≤ 0.05). Bars represent group averages and symbols represent individual animals.

The two-way ANOVAs for Radiation Dose X Trial for exploratory activity showed that activity was significantly greater on trial 1 compared to trial 3 of Habituation at the one month time point (F(1,41) = 83.02, *p* < 0.0001) and the six month time point (F(1,33) = 34.34, *p* < 0.0001), not differing among the groups (*p* = 0.695 and 0.337, respectively; see Figure 3). The two-way ANOVAs for the Recognition Test showed significant Radiation Dose X Trial interactions for the one month time point (F(2,41) = 4.348, *p* = 0.0194) and the six month time point (F(2,33) = 4.144, *p* = 0.0249). At one month, the 1 cGy group displayed significantly lower exploratory activity compared to the sham controls (*p* = 0.0257), but not the 10 cGy group (*p* = 0.2746). All groups displayed decreased activity on trial 3 and did not differ from the sham controls (all *p’*s ≥ 0.822). Given that the 1 cGy group had intact social recognition at this early time point, this decrease in exploratory activity was unrelated to this group’s performance. At six months, the 10 cGy group displayed greater activity on trial 3 of the Recognition Test compared to both the sham controls and the 1 cGy group (all *p’*s ≤ 0.0139), which demonstrates this group’s deficit is not simply a function of less exploratory activity. 

### 2.3. Ki67+ Cells in SVZ Unaffected by Radiation ^16^O Ion Exposure

**Figure 4:**
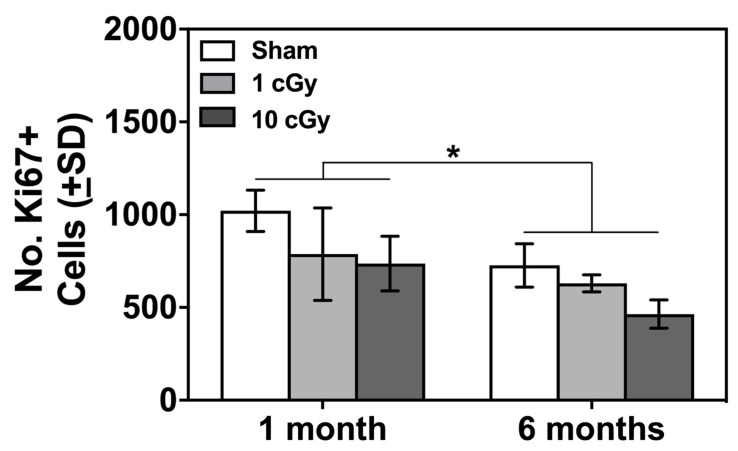
Mean number of Ki67+ cells in the subventricular zone (SVZ) of sham (*n* = 2–4), 1 cGy (*n* = 3–5), and 10 (*n* = 3–5) cGy-exposed rats. The number of Ki67+ cells was significantly greater when measured at one month following exposure compared to six months following exposure (* *p* < 0.05).

Cell proliferation in the SVZ in rodents has been linked to performance on olfactory-based learning and memory tests [26,27,28] and is negatively affected by radiation exposure [28,29]. The Radiation Dose X Time Following Radiation Exposure ANOVA revealed a significant main-effect of Time Following Radiation Exposure (F(1,17) = 5.9, *p* = 0.032, effect size: ω^2^ = 0.207). Collapsed across Radiation Dose, the number of Ki67+ cells in the SVZ was significantly greater at one month compared to six months following exposure (841.56 ± 73.79 vs. 571.33 ± 83.25, mean and SEM for one and six months, respectively; see Figure 4). This decrease corresponds to the different ages of the subjects when these analyses were completed (6–7 months old compared to 12–13 months old), which is indicative of an age-related decrease in cell proliferation in the SVZ that is unaffected by ^16^O ion exposure. 

### 2.4. Plasma KC/GRO (CXCL1) was Elevated at One Month Following ^16^O Ion Exposure in 1 cGy-Exposed Rats

**Figure 5:**
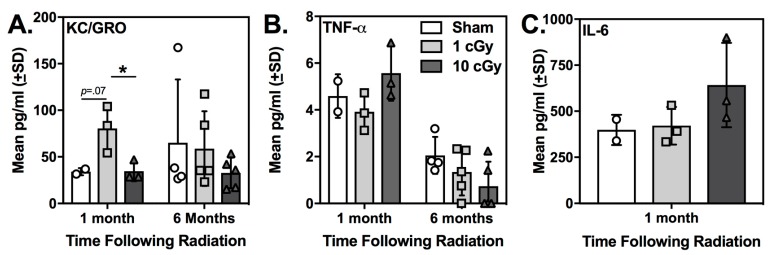
Plasma cytokine levels in sham control (*n* = 2–4), 1 cGy (*n* = 3–5), and 10 cGy (*n* = 3–5) exposed rats measured either one month or six months following exposure. (**A**) Mean plasma KC/GRO levels (pg/ml) were significantly greater in the 1 cGy group at one month following exposure compared to the 10 cGy group (* *p* = 0.05) and trended towards significance compared to the sham controls (*p* = 0.07). (**B**) Mean plasma TNF-α levels were unaffected by radiation exposure. (**C**) Mean plasma IL-6 levels did not differ among the groups at one month following exposure.

Pro-inflammatory cytokines and chemokines in plasma have the potential to be used as peripheral biomarkers for radiation-induced cognitive deficits in astronauts. For each cytokine that was detected (i.e., KC/GRO, TNF-α, and IL-6), a one-way ANOVA examining Radiation Dose was completed for each time point. KC/GRO (CXCL1) was detectable in plasma samples from sham and irradiated rats at both time points following radiation, but significant effects of radiation exposure were only evident at one month following exposure (see Figure 5). The one-way ANOVA for Radiation Dose was significant (F(2,7) = 6.661, *p* = 0.039, effect size: ω^2^ = 0.586). Tukey’s multiple comparisons test showed that plasma KC/GRO levels were significantly greater in the 1 cGy group compared to the 10 cGy group (*p* = 0.05); this difference approached significance for the comparison with the sham-irradiated rats (*p* = 0.071). These data suggest that KC/GRO could be an early indicator of intact social odor recognition following radiation exposure. At both one month and six months following exposure, no differences in TNF-α plasma levels were observed between irradiated groups and sham-irradiated animals (all *p’*s ≥ 0.173). No differences were found for plasma levels of IL-6 (*p* = 0.244) at one month following exposure. This cytokine was not detectable in any six-month plasma samples. Levene’s Test for homogeneity of variances was not significant for any comparison at either time point (one month: KC/GRO: *p* = 0.224; TNF-alpha: *p* = 0.618; IL-6: *p* = 0.159; six months: KC/GRO; *p* = 0.06; TNF-alpha: *p* = 0.587).

## 3. Discussion

Acute exposure to oxygen ions (^16^O) at 10 cGy, but not 1 cGy, impaired social recognition memory at one month following exposure, with all irradiated animals having impaired social recognition at six months following exposure. Further, plasma KC/GRO (CXCL1) levels were significantly elevated in the 1 cGy rats compared to the 10 cGy rats, which suggests that levels of this plasma cytokine may have potential for understanding the lack of early radiation-induced social recognition deficits. Importantly, ^16^O ion exposure did not affect the amount of time rats spent exploring the novel 1 odor beads during Habituation at either time point, indicating that radiation exposure did not impair social odor discrimination or olfactory processing. The ability of the irradiated rats to discriminate odors and show intact olfaction is also supported by our previous work [22] and the work of Krukowski et al. [8,30]. Both studies found no change in these measures following exposure to higher doses of ^16^O ions in the SORM and the Crawley three-chamber social interaction test. 

When comparing the current results to previous work [8,22,30], social recognition deficits appear to emerge over time in a dose-dependent fashion. First, ^16^O ion exposure (doses between 10–25 cGy) results in early and long-term impairments in social recognition in both male rats and male mice; this general finding across species could be indicative of the likelihood that exposure within this dose range would be deleterious for social processing in astronauts. However, higher exposure doses (40 cGy in mice) have not resulted in social recognition deficits [8]. Second, lower dose exposures appear to induce variable, unpredictable, and possibly even intermittent social recognition deficits that could reflect differences in detection and/or repair of radiation-induced damage [31,32,33]. For example, we previously reported impaired social recognition at one month following 5 cGy ^16^O (1000 MeV/n) ion exposure, with intact social recognition in this same group at six months following exposure. The present report found the opposite pattern of deficits following 1 cGy ^16^O (1000 MeV/n) exposure. This raises the possibility that the 1 cGy group sustained enough damage to develop impairments by six months, but not enough early damage to impair social processing or to initiate repair pathways, subsequently leading to late impairments in social recognition [31,34]. The increased plasma KC/GRO in this group at the early time point supports this hypothesis and while the sample size is small, it suggests early inflammatory changes in this group that could be indicative of intact social processing at early time points following radiation exposure. Importantly, early changes in plasma KC/GRO levels were not found in the 10 cGy group, which displayed impaired social recognition at this time point. These nonlinear responses are not uncommon for the effects of HZE ions and have been reported in several studies examining neurobehavioral function, brain vasculature, and the gut microbiome [31,35,36,37]. Interestingly, the gut microbiome was found to be more sensitive to 10 and 25 cGy ^16^O ions, with functional and metabolic changes significantly different from sham controls following exposure to these lower doses, but not following exposure to 1 Gy [31]. An intriguing hypothesis for future work is to determine if radiation-induced changes in the gut microbiome are significantly correlated with these dose- and time-dependent deficits in social recognition. To the best of our knowledge, the only other study examining low ^16^O exposure doses in social recognition tests in rodents used a simulated GCR exposure that included 20% of the total 0.5 Gy dose from ^16^O ions (~10 cGy from ^16^O ions, 594.4 MeV/n) and reported deficits in social recognition at 45 days following exposure [30], which supports this hypothesis that ^16^O ion doses within this range (10–25 cGy) induce predictable deficits in social recognition.

Further, this pattern of dose- and time-dependent social recognition deficits appears to be different in female subjects, indicating a possible sex difference following radiation exposure that needs to be further examined [8,26,38,39]. For example, female mice do not develop radiation-induced social recognition deficits following the multi-ion GCR-like exposure described above [30]. Further, following 5 Gy X-ray exposure, social odor recognition deficits in female mice were estrous-cycle dependent, with female mice in metestrus/dietstrus displaying intact social recognition that was not significantly different from female sham rats in metestrus/dietstrus or proestrus/estrus [26]. In contrast to the present study and previous reports [8,22,30], the Perez et al. [26] study investigating X-ray exposure used opposite sex odors for novel odor stimuli and thus mating and/or reproductive behaviors could play a role in these estrus cycle phase-dependent deficits. Nonetheless, estrus cycle could also be an important factor in the severity or etiology of social recognition deficits following HZE ion exposure and warrants further investigation, especially in light of the fact that crews on long-duration missions will most likely be of mixed gender.

Similar to our previous work with 5 and 25 cGy ^16^O (1000 MeV/n) ion exposure, the number of Ki67+ cells in the SVZ was equivalent in both irradiated and sham control rats when assessed at the six month time point. Further, no radiation-induced differences were found at the earlier time point. However, the number of Ki67+ cells decreased significantly between time points, with the older subjects (measured at six months, approximately 12–13 months old) displaying significantly less staining than the younger groups (measured at one month, approximately 7–8 months old). This significant age-related change in Ki67+ cell numbers in the SVZ is in agreement with previous work describing age-related decreases in cell proliferation and molecular markers indicative of the proliferative capacity of the SVZ in rodents [40]. Cell proliferation in the SVZ in rodents is associated with performance on olfactory-based learning and memory tests [26,27,28], with more robust olfactory memory and dishabituation responses for volatile odorants (e.g., (+)-carvone) found in mice with greater cell proliferation in the SVZ [41]. Several studies, however, have reported no change to olfaction and/or olfactory learning and memory after adult ablation of neurogenesis [42,43]. Interestingly, photon radiation exposure has been shown to decrease neurogenesis in both the subgranular zone of the dentate gyrus of the hippocampus and the SVZ in rats, with only neurogenesis in the SVZ showing long-term recovery [28,29]. It is possible that at the time points assessed in the current study, any radiation-induced decrease in cell proliferation had already recovered. Recovery of Ki67+ staining despite long-term neurobehavioral deficits and decreased survival of new neurons has been shown in the dentate gyrus following ^28^Si exposure [39], and similar results were found following ^12^C [44], as well. Further, the role of SVZ neurogenesis in social processing appears to be more robust in female rodents, with juvenile elimination of neurogenesis resulting in deficits in conspecific social interaction (i.e., age-matched females), pup retrieval in adulthood, and stress-related changes in social interaction [45]. More work is needed with female rodents exposed to HZE ions to determine if radiation induces changes in cell proliferation in the SVZ and if they are related to impairments in socially motivated tests of learning and memory in a sex-dependent and/or estrus cycle-dependent manner [26].

Recently, Krukowski and colleagues [8] found that lower CD8+ T cells in the periphery were significantly correlated with deficits in social recognition memory. In the present study, we found increases in KC/GRO in the 1 cGy-exposed rats at the one month time point only. Secretion of this cytokine is associated with angiogenesis, inflammation, and inhibition of oligodendrocyte precursors in the CNS, but a neuroprotective role for KC/GRO has been reported [46]. Interestingly KC/GRO has also been shown to be upregulated following both low and high LET irradiation [47,48]. For example, following exposure to 10 cGy ^56^Fe ions, expression of KC/GRO was upregulated in human thyroid epithelial cells; however, it is unclear how these cellular responses relate to neurobehavioral performances assessed in rodents. It is possible that elevated plasma levels of KC/GRO are associated with a lack of early radiation-induced social processing deficits, since this cytokine was only elevated in the 1 cGy group (i.e., the irradiated group showing no early deficits). While these data suggest a possible role of KC/GRO in early responses to radiation, the sample size in the current study was small and more work is needed to confirm these findings in larger cohorts. Further, exactly why this cytokine was elevated in only the 1 cGy group and not the 10 cGy group is unknown, but this finding does mimic the previous study in mice where only the 25 cGy-exposed subjects (and not the 40 cGy-exposed subjects) displayed significant decreases in CD8+ cells and social behavior deficits [8]. These results could be related to the previously described nonlinear responses following low dose radiation exposure, which could also explain some of the variability in the cytokine levels at the six month time point, including plasma KC/GRO levels. Given the complexities of pro- and anti-inflammatory signaling after radiation, these results demonstrate that more work is needed to understand if peripheral cytokines are reliable biomarkers for neurobehavioral deficits and what mechanism(s) mediate these effects.

Rodents are social animals that often live in groups and engage in reciprocal interactions with conspecifics [49]. However, social interactions that result in subordination are stressful for rodents [50]. In the present study, subjects were single housed and it is possible that single housing a social species could result in deleterious effects on the brain and behavior [50]. While solitary housing has been shown to induce behavioral changes in rodents, it is unlikely these effects are occurring in the present study for several reasons. First, all subjects in the present study have access to enrichment toys and nesting material (described in the Methods) throughout the entire experiment. Access to these items is often part of an enriched environment and has been shown to have positive effects on behavioral, cellular, and molecular endpoints in rodents. Second, the sham control animals in the present study display intact social recognition memory at both time points tested and they are housed under the exact same conditions and live in the same vivarium as the irradiated groups. Finally, the fact that radiation-induced deficits varied with dose and time suggests that the design of the current experiment was sensitive enough to identify the effects of radiation on social recognition in our singly housed animals. 

Overall, there seems to be a specific window in which the dose of ^16^O ion exposure that causes long-term, persistent social recognition deficits. Our results suggest that plasma cytokines, like KC/GRO, have the potential to be used as biomarkers of social behavior following ^16^O ion exposure. These results have important implications for long duration missions and demonstrate that behaviors related to social processing could be negatively affected by HZE ion exposure. 

## 4. Methods

### 4.1 Subjects and Apparatus

Male Long-Evans rats (*n* = 44, Envigo, East Millstone, NJ, USA) were received in the laboratory at 10–12 weeks of age and irradiated at approximately 5–6 months of age. Rats were singly housed in individual plastic cages and maintained on a 12:12 h reversed light-dark schedule (lights on at 1800-h) and at an ambient temperature of 23 °C for the duration of the experiment. Rats were maintained at 90% of their free-feeding weights by being fed measured amounts of chow each day after they performed another behavioral test daily (not reported here). No other testing was conducted on the three days of the SORM test at each time point. Rats were tested and fed during the active phase of their cycle (i.e., during the lights off period). Water was freely available in the home cage. All rats had continuous access to enrichment toys (wooden blocks, Chewy Nylabones, and polycarbonate tunnels; BioServ, Flemington, NJ, USA) when they arrived in the laboratory and for the duration of the experiment. All behavioral testing was performed in the dark phase between 0900–1300 under red light conditions. Laboratory animal care was conducted according to Public Health Service (PHS) Policy on the Humane Care and Use of Laboratory Animals, and the Institutional Animal Care and Use Committee of the Johns Hopkins University (RA17M78) approved all procedures on 23 March 2017. Johns Hopkins also maintains accreditation of their program by the Association for the Assessment and Accreditation of Laboratory Animal Care (AAALAC).

### 4.2. Radiation Procedures

When rats were approximately 5–6 months old, they were exported to Brookhaven National Laboratory (BNL) seven days prior to the scheduled exposure day. All animals were exposed to whole body radiation on the same day and then returned to Johns Hopkins for follow-up testing 10 days post-irradiation. During exposures, rats were placed in individual plastic holders, and then acutely exposed to ^16^O ions (1000 MeV/n) generated at the NASA Space Radiation Laboratory (NSRL) facility at BNL. ^16^O ions of this energy have a mean range in water of approximately 81 cm with an average linear energy transfer (LET) of 14 keV/*μ*m. Target exposure levels were 1 and 10 cGy (*n* = 16 rats/dose); actual delivered doses varied by no more than 0.1 cGy relative to each target dose. Dose rates were 1.78–4.09 cGy/min. The control group (*n* = 13 rats) was sham irradiated (i.e., shipped to BNL, placed in the plastic holder, but not brought into the beam line). 

### 4.3. Social Odor Recognition Memory Test (SORM)

The SORM test consisted of three phases: Familiarization, Habituation, and the Recognition Test, each separated by 24 h, and were conducted in a manner identical to our previous report [22]. All trials were video recorded under red light using an infrared night vision digital video camera (Hausbell 302S FHD Camcorder, Rosemead, CA, USA) and scored offline with The Observer XT software (Noldus, Leesburg, VA, USA) by experimenters blinded to the odor-impregnated bead (see below) and rat irradiation conditions. Beads and rats were number coded for behavioral scoring of video. The three-day testing period occurred at one and six months following exposure. Different novel social odors from conspecifics housed in a different vivarium were used for each test period. The phases below started seven days after the last cage cleaning to provide for distinctive conspecific odor cues [22,23].

### 4.4. Familiarization

During Familiarization, eight 2.5-cm round, unfinished wooden beads (CraftParts Direct) were introduced into each rat’s home cage to acquire the odor of the rats and serve as familiar odors (beads F1, F2, and F3) for the subsequent phases. Wooden beads were also placed into the cages of separate ‘‘odor donor’’ rats to provide novel 1 (N1) and novel 2 (N2) bead odors for the subsequent test phases. 

### 4.5. Habituation

At 24 h, after Familiarization and 1 h before testing, all wooden beads were removed from the rats’ cages and placed in individual plastic bags with bedding from each rat. For testing, three familiar odor beads and one novel 1 odor bead (N1) were introduced into each rat’s home cage. The order of the four beads was randomly altered for each rat. Beads were placed in a row in the center of each rat’s cage. Rats were exposed to the four beads for three 1 min trials separated by 1 min inter-trial intervals (during which time the beads were removed from the cage). During each 1 min trial, rats were allowed to freely explore the beads. Upon initial contact with any of the four beads, a 1 min timer was started and the rats’ behavior was video-recorded for subsequent analysis. A trial was scored as incomplete if rats failed to make contact with a bead after two minutes. All beads from this phase were discarded after use.

### 4.6. Recognition Test

In a manner similar to Habituation, rats were presented with four beads 24-h later: two familiar odor beads, one novel odor 1 (N1) bead (the odor experienced during the habituation phase 24 h earlier) and one novel odor 2 (N2) bead (an unfamiliar, novel odor not experienced previously), with the order of the four beads randomly altered for each rat identical to the methods described above for Habituation.

### 4.7. Immunohistochemistry

One week after the one-month or six-month memory test, rats (*n* = 2–5/group) were euthanized by decapitation and brains removed. The left hemispheres were immersed in 30% sucrose for 3 h at 4 °C, after which they were embedded in TissueTek and snap frozen in liquid nitrogen and stored at –80 °C until processed. Sections (20 μm) of the subventricular zone (SVZ) from 0.5–1.4-mm bregma were collected for the sham-, 1 and 10 cGy-irradiated groups at both time points. Sections were thaw-mounted onto APS-coated slides and fixed with 0.5% paraformaldehyde for 5 min. Sections were then washed with PBS (3 × 5 min) and incubated overnight with the primary antibody, anti-Ki67 (1:300, ab15580; Abcam, Cambridge, MA, USA) at 4°C with gentle agitation in a humidity chamber. Sections were washed with PBS (3 × 5 min) and incubated for 1 h with the secondary antibody, goat anti-rabbit IgG H and L (Alexa Fluor 488), at 1:600 (ab150077; Abcam). A final wash with PBS (3 × 5 min) was completed and then slides were coverslipped with Vectashield with DAPI (Vector Laboratories, Burlingame, CA, USA). Five slices, separated by 100 μm per slice, were processed per animal. Cell counts from the five slices were summed for each animal to compute a group average. Slides were viewed using an Olympus BX61 with Roper/Photometrics Coolsnap HQ (Roper Technologies, Tucson, AZ, USA) in the Johns Hopkins University School of Medicine Institute for Basic Biomedical Sciences Microscope Facility. Observers blinded to each rat’s radiation condition counted Ki67+ cells using SlideBook Reader (v6.0.4, Intelligent Imaging Innovations, Inc., Denver, CO, USA). 

### 4.8. Rat V-Plex Proinflammatory Cytokine Panel

Cytokine assays were completed using the Meso Scale Diagnostics (MSD) V-Plex Proinflammatory Panel 2 Rat Kit (Cat. No. K15059D, MSD, Rockville, MD, USA), which allows for the simultaneous measurement of IFN-γ, IL-10, IL-13, IL-1β, IL-4, IL-5, IL-6, KC/GRO, TNF-α. Following euthanasia at the time points described above, trunk blood was collected into EDTA-coated microcentrifuge tubes. The samples were centrifuged at 3000 rpm for 15 minutes. The supernatant (plasma) was then pipetted off into smaller aliquots, snap frozen in liquid nitrogen, and stored at –80 °C until processed. For processing, plasma aliquots were thawed and each sample was run in duplicate. 150ul of Blocker H was added to each well on the 96-well plate and incubated at room temperature for 1 h with gentle shaking. The plate was then washed three times with PBS with Tween-20 (0.5%; PBS-T) wash buffer. Next, 50ul of each sample (diluted in Diluent 42) or calibrator were added to each well, and again incubated at room temperature for 2 h with gentle shaking. The plate was then washed three times with PBS-T wash buffer. 25ul of detection antibody solution was added to each well. The plate was then incubated at room temperature for 2 h with gentle shaking. Finally, the plate was washed three times with PBS-T wash buffer. 150ul of MSD Read Buffer T was added to each well, and the plate was read immediately by the Sector Imager (MSD, Rockville, MD, USA). 

### 4.9. Data Analysis

Time spent exploring [e.g., sniffing, whisking near (within 1 cm), manipulating beads with paws and/or mouth, licking beads] each wooden bead (familiar beads, N1 and N2) was measured in seconds for each 1 min trial for both the habituation and recognition test phases at one month and six months following irradiation. Total raw seconds of exploring for each bead were summed separately for each trial of the habituation and recognition test phases at each time point for each bead type for each rat. Percentage time was calculated for each bead: time exploring bead/total time exploring all beads, e.g., N1/(N1 + F1 + F2 + F3), identical to our previous work with ^16^O ions [22]. For Habituation, the first 1 min trial for each post-irradiation time point was used to determine preference for the N1 odors during this phase; greater exploration of the N1 odor compared to the familiar odors on this trial supported the salience of the novel odor stimulus and the statistical significance of this effect was assessed with within-subject t-tests. A significant reduction in exploration time between the N1 odor on the first 1 min trial and the last (third) 1 min trial during Habituation, supported the fact that rats displayed habituation to the N1 odors, which was also assessed with a within-subject t-test. For the Recognition Test, the first 1 min trial for each post-irradiation time point was used to determine 24-h recognition memory for the N1 odor from the previous day. Memory for the N1 odor was demonstrated by significantly greater time spent exploring the N2 odor, compared to the N1 odor, on the first trial of the Recognition Test. Statistical significance was assessed with a within-subjects t-test for each group. Effect sizes were also determined using Cohen’s d. To compare the different groups at each time point on the Recognition Test, a discrimination index (DI) was also calculated using total exploration time summed for the three trials separately for the N2, N1 and familiar beads as follows: DI = [(N2 – N1)/(N2 + N1 + F1 + F2)] × 100. Discrimination index values greater than zero demonstrate preference for the N2 odor bead, whereas values less than zero show preference for the N1 odor bead. In addition, DI values equal to zero demonstrate that the N1 and N2 odor beads were explored to a similar degree. A one-way ANOVA followed by Dunnett’s multiple comparisons test was used to compare the discrimination index of the sham-irradiated rats with the 1 or 10 cGy groups at each time point. Total exploration activity on Trials 1 and Trials 3 of Habituation and the Recognition Test at each time point were calculated separately for each animal and presented as a group mean for each trial each time point. Separate two-way ANOVAs (Radiation Dose X Trial) followed by Tukey’s multiple comparisons tests were used to compare total activity among the groups on each trial at each time point. Alpha was set to 0.05 for all ANOVAs and post-hoc tests. 

For Ki67+ staining, mean cell counts for each group were compared using a two-way ANOVA (Radiation Dose X Time Following Radiation Exposure). For each detectable cytokine, a Radiation Dose X Time Following Radiation Exposure two-way ANOVA was used to determine significant changes in plasma cytokine levels between the exposure groups and time points. Significant interactions were followed by Tukey’s multiple comparisons test. Effect sizes were also calculated for each ANOVA (omega squared) and each group comparison (Hedge’s *g* for small sample sizes). For all tests, alpha was set to 0.05. Homogeneity of variances was assessed with the Levene’s Test. All statistical analyses were completed with GraphPad Prism version 6.0e (GraphPad Software LaJolla, CA, USA) or SPSS version 24 (IBM Corp., Armonk, NY, USA).

## Figures and Tables

**Table 1:** Novel 1 odor (N1) exploration during Habituation at one month post-exposure.

Group	N1 Odor Trial 1 (s)	Familiar Odors Trial 1 (s)	*t* value	*p* value	N1 Odor Trial 3 (s)	*t* value	*p* value
Sham	14.9 ± 2.85	3.63 ± 0.38	3.959	0.002	5.12 ± 1.95	2.790	0.024
1 cGy	16.01 ± 2.8	3.65 ± 0.43	4.092	0.001	4.56 ± 1.41	4.607	<0.001
10 cGy	14.54 ± 2.85	2.88 ± 0.34	4.095	0.001	4.22 ± 2.03	4.494	0.001

Mean (±SEM) exploration time in seconds for the N1 odor on the first and third trial and the average of the three familiar odor beads on the first trial. Sample size: *n* = 12–16 rats per group.

**Table 2:** Novel 1 odor (N1) exploration during Habituation at six months post-exposure.

Group	N1 Odor Trial 1 (s)	Familiar Odors Trial 1 (s)	*t* value	*p* value	N1 Odor Trial 3 (s)	*t* value	*p* value
Sham	19.40 ± 3.80	4.89 ± 0.49	3.637	0.005	4.50 ± 2.10	3.140	0.012
1 cGy	22.57 ± 3.24	5.14 ± 0.72	4.669	0.001	7.78 ± 2.07	4.175	0.001
10 cGy	23.28 ± 2.67	4.16 ± 0.52	6.463	<0.001	12.8 ± 3.28	2.581	0.024

Mean (±SEM) exploration time in seconds for the N1 odor on the first and third trial and the average of the three familiar odor beads on the first trial. Sample size: *n* = 10–13 rats per group.
